# Efficacy and safety of glecaprevir/pibrentasvir in HCV-infected Japanese patients with prior DAA experience, severe renal impairment, or genotype 3 infection

**DOI:** 10.1007/s00535-017-1396-0

**Published:** 2017-10-20

**Authors:** Hiromitsu Kumada, Tsunamasa Watanabe, Fumitaka Suzuki, Kenji Ikeda, Ken Sato, Hidenori Toyoda, Masanori Atsukawa, Akio Ido, Akinobu Takaki, Nobuyuki Enomoto, Koji Kato, Katia Alves, Margaret Burroughs, Rebecca Redman, David Pugatch, Tami J. Pilot-Matias, Preethi Krishnan, Rajneet K. Oberoi, Wangang Xie, Kazuaki Chayama

**Affiliations:** 1 Department of Hepatology, Toranomon Hospital, Tokyo, Japan; 2 Department of Internal Medicine, St. Marianna University School of Medicine, Kawasaki, Japan; 3 Department of Medicine and Molecular Science, Gunma University Hospital, Maebashi, Japan; 4 Department of Gastroenterology, Ogaki Municipal Hospital, Gifu, Japan; 5 Department of Internal Medicine, Nippon Medical School, Tokyo, Japan; 6 Department of Human and Environmental Sciences, Kagoshima University Hospital, Kagoshima, Japan; 7 Department of Gastroenterology and Hepatology, Okayama University Graduate School of Medicine, Dentistry, and Pharmaceutical Sciences, Okayama, Japan; 8 The First Department of Internal Medicine, Faculty of Medicine, University of Yamanashi, Yamanashi, Japan; 9 AbbVie Inc., North Chicago, IL USA; 10 Department of Gastroenterology and Metabolism, Institute of Biomedical and Health Sciences, Hiroshima University Hospital, Hiroshima, Japan

**Keywords:** Cirrhosis, Glecaprevir, Pibrentasvir, Renal failure, Special population

## Abstract

**Background:**

Once-daily, orally administered, co-formulated glecaprevir (NS3/4A protease inhibitor) and pibrentasvir (NS5A inhibitor) (G/P) demonstrated pangenotypic activity and high sustained virologic response (SVR) rates in studies outside Japan. Here we report safety and efficacy in a subset of Japanese patients with chronic HCV infection who received G/P 300/120 mg in a phase 3, open-label, multicenter study (CERTAIN-1).

**Methods:**

This analysis focuses on three difficult-to-treat subgroups: HCV GT1/2-infected patients who failed to achieve SVR after treatment with a direct acting antiviral (DAA)-containing regimen; GT1/2-infected patients with severe renal impairment (estimated glomerular filtration rate < 30 mL/min/1.73 m^2^); and GT3-infected patients. Patients in the renal impairment and GT3 cohorts were treatment-naive or interferon treatment-experienced. Noncirrhotic GT1/2-infected, DAA-naïve patients in the renal impairment cohort received G/P for 8 weeks; all other patients were treated for 12 weeks. Primary outcome was SVR (HCV RNA < 15 IU/mL) 12 weeks post-treatment (SVR_12_).

**Results:**

The study enrolled 33 GT1/2-infected patients who failed previous DAA treatment (four with cirrhosis); 12 GT1/2-infected patients with severe renal impairment (two with cirrhosis); and 12 GT3-infected patients (two with cirrhosis). SVR_12_ was achieved by 31/33 (93.9%), 12/12 (100%), and 10/12 (83.3%) patients, respectively. One serious adverse event (fluid overload, not related to G/P) occurred in a patient on chronic intermittent hemodialysis.

**Conclusions:**

G/P achieved high SVR_12_ rates and was well tolerated in three difficult-to-treat patient subgroups with limited treatment options in Japan (DAA-experienced patients, patients with severe renal impairment, and GT3-infected patients). These results support the potential suitability of this regimen for these special populations in Japan.

**Electronic supplementary material:**

The online version of this article (doi:10.1007/s00535-017-1396-0) contains supplementary material, which is available to authorized users.

## Introduction

There are 1.5 million individuals estimated to be infected with hepatitis C virus (HCV) in Japan [1–4]; of those, approximately 67% are infected with genotype 1 (GT1), 30% with GT2, and 3% with other genotypes [5]. When left untreated, HCV infection can lead to liver cirrhosis, hepatocellular carcinoma, and end-stage liver disease [6, 7]. Combinations of direct acting antiviral agents (DAAs) such as ledipasvir/sofosbuvir, daclatasvir + asunaprevir, ombitasvir/paritaprevir/ritonavir, elbasvir/grazoprevir, or asunaprevir/daclatasvir/beclabuvir have demonstrated high rates of sustained virologic response (SVR) in Japanese patients with chronic GT1 HCV infection [8–10], and sofosbuvir + ribavirin (RBV) and ombitasvir/paritaprevir/ritonavir + RBV are approved for treatment of HCV GT2. The recent approval of sofosbuvir + RBV for patients infected with HCV genotypes other than GT1 and GT2 has also helped to address an unmet medical need for these patients in Japan. However, treatment options remain limited for some HCV-infected patients currently considered difficult to cure, including GT1- and GT2-infected patients who failed prior DAA treatment, GT2–6-infected patients with severe renal impairment (CKD stages 4 and 5), and those with GT3 infection.

The Japan Society of Hepatology (JSH) guidelines for the management of hepatitis C virus infection recommend ledipasvir/sofosbuvir for HCV GT1-infected and sofosbuvir + RBV for GT2-infected patients without severe renal impairment who previously failed to achieve SVR with a prior protease inhibitor (PI) + pegylated interferon (pegIFN) + RBV regimen [11]. For GT1-infected patients who failed previous PI + NS5A-inhibitor therapy, retreatment with simeprevir + pegIFN + RBV, or ledipasvir/sofosbuvir are recommended options dependent on patient and viral characteristics. Since there is insufficient evidence on the impact of DAA-resistance in ledipasvir/sofosbuvir failures on future treatment, the JSH guideline also suggests considering anticipating new drug development as an option [11].

There are currently no approved treatment options for GT2–6-infected patients with severe renal impairment in Japan. IFN-free DAA combination regimens of sofosbuvir + RBV and ombitasvir/paritaprevir/ritonavir + RBV are recommended for some HCV GT2-infected patients. However, sofosbuvir is contraindicated for use in patients with severe renal dysfunction regardless of genotype. Additionally, RBV, is contraindicated in patients with moderate or severe renal impairment and should be avoided in patients with severe cardiac conditions, leaving GT2-infected patients with these comorbidities without an IFN-free treatment option.

For GT3 infected patients, treatment options include pegIFN + RBV, which produces low SVR rates (68%) and has poor tolerability, particularly in the elderly and patients with comorbidities such as psychiatric illness, autoimmune disorders, and cytopenias [12–14], and the recently approved DAA-based regimen sofosbuvir + RBV for 24 weeks, which produces an SVR rate of 85% [15]. Both of these regimens contain RBV and are thus subject to the contraindications and associated with adverse events typical of RBV (e.g., hemolytic anemia and pruritus).

Hence, there is an unmet need for a single potent, once a day, well-tolerated, RBV- and IFN-free pangenotypic regimen with high efficacy in the majority of GT1 or GT2 HCV-infected, DAA-naive Japanese patients, including those with stages 4 or 5 CKD, as well as other special populations with limited treatment options, that could help simplify and improve the current standard of care in HCV-infected Japanese patients.

Glecaprevir (GLE, formerly ABT-493, identified by AbbVie and Enanta), an NS3/4A protease inhibitor co-formulated with pibrentasvir (PIB, formerly ABT-530), an NS5A inhibitor, is currently being evaluated as a pangenotypic regimen (G/P) for patients with chronic HCV infection including “difficult-to-treat” subgroups such as patients with compensated cirrhosis, chronic renal failure, or those who failed to respond to previous DAA-based therapy. Preclinical studies have demonstrated that this combination regimen has a high barrier to resistance and potency against common NS3 and NS5A polymorphisms [16]. High efficacy of G/P in various patient populations over short treatment durations compared to currently recommended treatments has been demonstrated outside Japan, including in patients who have been previously considered difficult-to-treat. Here we describe the safety and efficacy of G/P in GT1 or GT2 HCV-infected patients who failed to achieve SVR with prior DAA-treatment or those with severe renal impairment including those with compensated cirrhosis, and in GT3 HCV-infected patients.

## Methods

### Study design

**Fig. 1:**
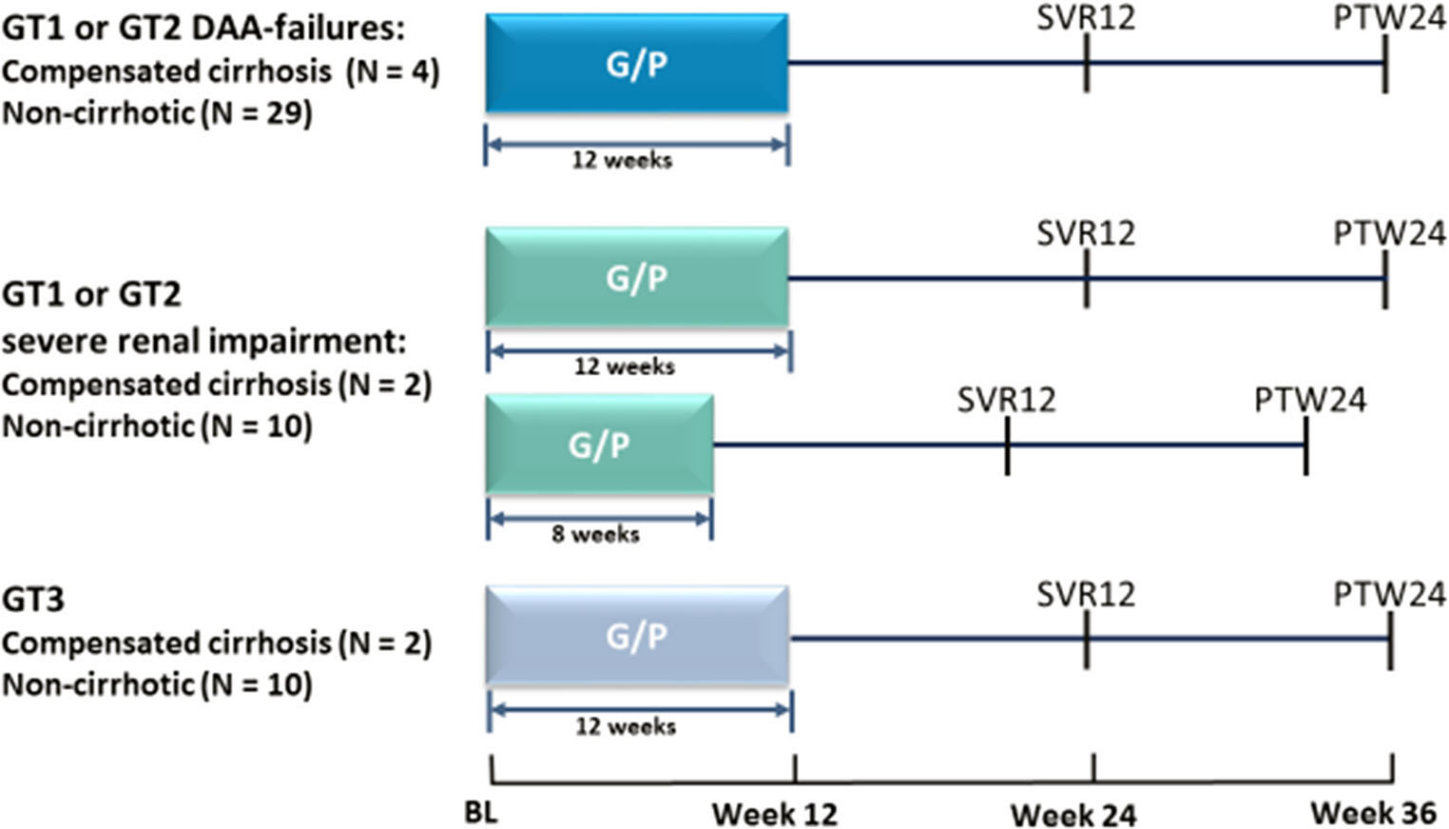
Study design for special populations of patients enrolled in the CERTAIN-1 substudy 2. *BL* baseline, *PTW* post-treatment week, *SVR* sustained virologic response

CERTAIN-1 is a phase 3, open-label, multicenter study assessing the safety and efficacy of G/P (300/120 mg) once daily in Japanese patients with HCV infection. Results from substudy 1 where HCV GT1-infected patients without cirrhosis were treated for 8 weeks with G/P or 12 weeks with ombitasvir/paritaprevir/ritonavir, as well as patients in substudy 2 with HCV GT1 and GT2 infection and compensated cirrhosis treated with G/P for 12 weeks have been reported elsewhere [17, 18]. Here we report on the remainder of patients in substudy 2 who belong to four special populations: HCV GT1- or GT2-infected patients who failed prior DAA-treatment including patients with compensated cirrhosis, GT1 or GT2 HCV-infected patients with severe renal impairment and compensated cirrhosis, GT3 HCV-infected patients who were treatment-naive or IFN treatment-experienced, all of whom received treatment with G/P for 12 weeks, and patients with GT1 or GT2 HCV infection with severe renal impairment without cirrhosis who received treatment for 8 weeks. In this report we combine the results from patients with severe renal impairment with or without compensated cirrhosis. Figure 1 shows the study design.

All patients provided written, informed consent to participate, and the study was conducted consistent with the ethical guidelines of the Declaration of Helsinki and the International Conference on Harmonisation Good Clinical Practice Guidelines. The study was approved by an institutional review board of each study site prior to the initiation of any screening or study-specific procedures. Supporting Figure 1 shows patient disposition.

### Patients

Patients were screened from February 22, 2016 to June 1, 2016 at 62 study sites in Japan. Adults 18 years or older were eligible for enrollment if they had chronic HCV GT1 to GT6 infection documented by positive anti-HCV antibody test and a plasma HCV RNA level ≥ 1,000 IU/mL at the time of screening. Plasma samples for HCV genotype and subtype determination were collected at screening and assessed using the appropriate assay by the central laboratory.

Presence or absence of compensated cirrhosis was determined by liver biopsy, Fibroscan, FibroTest/aspartate aminotransferase to platelet ratio index, or discriminant score test. Additional details are listed in the Supporting Information. The study population comprised patients with and without compensated cirrhosis and HCV GT1 or GT2 infection who failed to achieve SVR with at least 1 approved, commercially available HCV DAA-containing regimen ≥ 2 months prior to screening were enrolled as prior DAA-failures. In addition, HCV GT1 or GT2 infected patients with severe renal impairment (with or without compensated cirrhosis) and an estimated glomerular filtration rate (eGFR) < 30 mL/min/1.73 m^2^ [using the MDRD method modified for Japanese population: eGFR_J_ = 194 × Serum Creatinine^−1.094^ × Age^−0.287^ × 0.739 (if female)], including patients with end-stage renal disease requiring treatment with chronic intermittent hemodialysis were also enrolled.

Patients were excluded from this study if they had a positive test result for hepatitis B surface antigen or anti-human immunodeficiency virus antibody, or had any cause of liver disease other than chronic HCV infection. Patients were also excluded if they had any current or past clinical evidence of Child–Pugh B or C classification or clinical history of decompensated liver disease such as ascites, hepatic encephalopathy or variceal bleeding, any clinically significant abnormalities or co-morbidities that make the patient an unsuitable candidate for this study in the opinion of the investigator, evidence of hepatocellular carcinoma upon imaging, or abnormal screening laboratory results as listed in Table 1.

**Table 1:** Abnormal laboratory results exclusion criteria for patients without cirrhosis and patients with compensated cirrhosis

Assessment	No cirrhosis	Compensated cirrhosis
Serum albumin, g/dL	< LLN	< 2.8
INR	≥ 1.2	≥ 1.8
Hemoglobin, g/dL	< 10	< 10
Platelet count, cells/mm^3^	< 90,000	< 50,000

*INR* international normalized ratio, *LLN* lower limit of normal

### Study assessment

Virologic response was assessed by plasma HCV RNA levels at various time points. Samples were collected at the screening visit, Day 1 visit and treatment Weeks 1, 2, 4, 8 (and 12 for patients with 12-week treatment duration) and post-treatment Weeks 2, 4, 8, 12, and 24. Plasma HCV RNA levels were determined for each sample collected by the central laboratory using the Roche COBAS^®^ AmpliPrep/COBAS^®^ TaqMan HCV Quantitative Test, v2.0. The lower limit of detection (LLOD) and lower limit of quantification (LLOQ) for this assay (regardless of genotype) were both 15 IU/mL. The efficacy endpoints for the three cohorts reported in this analysis are the percentage of patients achieving SVR_12_, the percentage of patients with virologic failure during treatment and relapse post treatment.

Next-generation sequencing was performed on samples collected from all patients at baseline, and presence of HCV baseline polymorphisms in NS3 and NS5A was evaluated using a 15% detection threshold. For patients who experienced virologic failure, treatment-emergent substitutions in NS3 and NS5A relative to the patient’s baseline HCV sequence were analyzed. Blood samples for pharmacokinetic (PK) assessment of the study drugs were collected from patients during each study visit. Patients consenting to intensive pharmacokinetic sampling had samples drawn at the Study Day 1 (at 2, 4, and 6 h post-dose) and at Week 4 visit at hour 0 (before study drug administration) and 2 and 4 h post-dose. Plasma concentrations for GLE and PIB were summarized as steady-state trough levels (C_trough_) based on binning of PK samples fall in the interval of 22–26 h after dosing. Plasma concentrations were determined using a validated assay at AbbVie.

Treatment-emergent adverse events (TEAEs) and laboratory tests were assessed and recorded throughout the treatment period until 30 days following discontinuation of study treatment. After the 30 day post treatment visit until the post-treatment Week 24 visit only spontaneously reported serious AEs (SAEs) were collected. All AEs were graded according to Common Terminology Criteria for Adverse Events (CTCAE), version 4.0.

### Statistical analysis

Analysis of these special populations was conducted after all enrolled patients completed the post-treatment Week 12 visit. Efficacy, safety, and demographic analyses were performed on all patients in the intent-to-treat (ITT) population, defined as all patients who received at least one dose of study drug. The number and percentage of patients achieving SVR_12_, with on-treatment virologic failure and post-treatment relapse and a 2-sided 95% confidence interval (CI) using the Wilson’s score method were computed. Descriptive statistics, such as the number of observations, mean, and standard deviation were provided for continuous variables.

## Results

### Baseline patient demographics and characteristics

A total of 57 patients were included in this analysis; 33 in the failed prior DAA treatment cohort, 12 in the severe renal impairment cohort, and 12 in HCV GT3-infected cohort. Of those, 61, 50, and 50% were female in the three cohorts, respectively. The mean (SD) HCV RNA at baseline was 6.0 (0.5), 5.8 (1.2), and 6.2 (0.7) log_10_ IU/mL, respectively. The median age in the three cohorts was 67, 69, and 57 years, respectively. Compensated cirrhosis was present in 12% of DAA-experienced patients, 17% of those with severe renal impairment, and 17% in those with GT3 infection. Among DAA-experienced patients, most had also failed a prior DAA-free, IFN-containing regimen, and 91% had failed prior daclatasvir + asunaprevir treatment. Detailed patient demographic and baseline characteristics are outlined in Table 2.

**Table 2:** Baseline demographics and disease characteristics of special-population patients enrolled in CERTAIN-1

Characteristic	DAA-failures^a^ *N* = 33	Severe renal impairment^b^ *N* = 12	HCV GT3-infected^c^ *N* = 12
Female, *n* (%)	20 (61)	6 (50)	6 (50)
Age, median (range), years	67 (53–80)	69 (54–78)	57 (23–70)
Age, *n* (%)			
< 65	12 (36)	4 (33)	9 (75)
≥ 65 and < 75	16 (49)	6 (50)	3 (25)
≥ 75	5 (15)	2 (17)	0
BMI, mean ± SD, kg/m^2^	24.0 ± 3.5	22.2 ± 3.6	23.5 ± 3.9
HCV genotype, *n* (%)			
1	32 (97)	3 (25)	NA
2	1 (3)	9 (75)	NA
3	NA	NA	12
eGFR, mL/min/1.73 m^2^, *n* (%)			
≥ 30	33 (100)	1 (8)	12 (100)
< 30	0	11 (92)	0
On hemodialysis	–	4 (33)	–
IL28B non-CC genotype, *n* (%)	17 (52)	4 (33)	2 (17)
HCV RNA, mean ± SD, log_10_ IU/mL	6.0 ± 0.5	5.8 ± 1.2	6.2 ± 0.7
HCV RNA ≥ 800,000 IU/mL, *n* (%)	21 (64)	6 (50)	8 (67)
Treatment-naive, *n* (%)	0	9 (75)	7 (58)
Treatment-experienced, *n* (%)	33 (100)	3 (25)	5 (41)
IFN-based	11 (33)^d^	3 (100)	5 (100)
DAA-based	33 (100)	0	0
DCV + ASV	30 (91)	–	–
PegIFN + RBV + SMV	2 (6)	–	–
SOF + RBV	1 (3)	–	–
Compensated cirrhosis			
No	29 (88)	10 (83)	10 (83)
Yes	4 (12)	2 (17)	2 (17)

*ASV* asunaprevir, *DCV* daclatasvir, *NA* not applicable, *PegIFN* pegylated interferon, *RBV* ribavirin, *SMV* simeprevir, *SOF* sofosbuvir

^a^Included GT1- or GT2-infected patients with or without compensated cirrhosis who failed prior DAA treatment

^b^Included GT1- or GT2-infected patients with or without compensated cirrhosis and with severe renal impairment

^c^Included GT3-infected patients with or without compensated cirrhosis

^d^IFN-based treatment before DAA-based therapy

### Efficacy outcomes

**Fig. 2:**
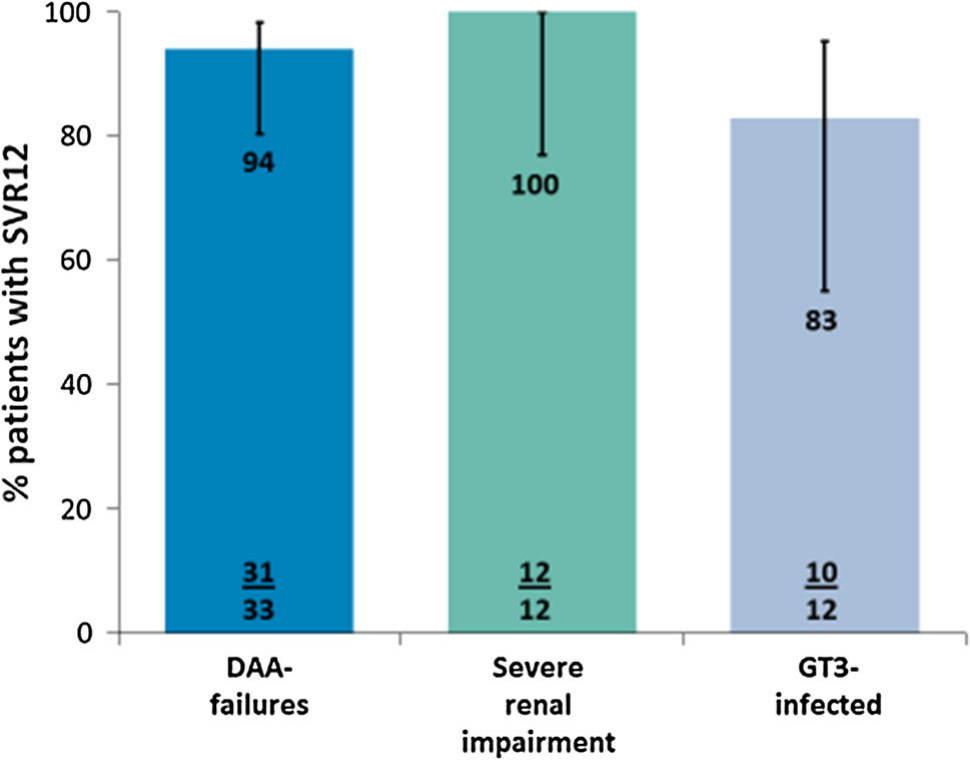
SVR_12_ rates for each arm in the ITT population. The error bars represent the 95% CI. Bar colors match SVR12 data of special patient populations to the study design (Fig. 1)

SVR_12_ was achieved by 31/33 (93.9%; 95% CI 80.4–98.3%) patients who failed to achieve SVR with at least 1 prior DAA-containing regimen; including three of four patients with cirrhosis, 12/12 (100%; 75.8–100%) patients with severe renal impairment, including the two patients with cirrhosis; and 10/12 (83.3%; 95% CI 55.2–95.3%) GT3-infected patients including the two patients with cirrhosis. Among those with prior DAA failure, the SVR_12_ rates were 93.3% (28/30) in patients who failed prior daclatasvir + asunaprevir, and 100% in patients who failed prior simeprevir + pegIFN + RBV (2/2) or sofosbuvir + RBV (1/1). One patient who failed prior DAA-treatment experienced on-treatment virologic failure and the other patient relapsed at post-treatment Week 4. During the study, two noncirrhotic GT3 infected patients failed to achieve SVR_12_. One subject was treatment–naive who relapsed at post-treatment Week 12. The plasma concentrations of this subject were comparable to other GT3 subjects who achieved SVR_12_ (pharmacokinetic data not shown). The second GT3-infected subject who failed to achieve SVR_12_ had received prior interferon-based treatment and relapsed at post-treatment Week 2. The plasma concentrations of this subject were substantially low for both GLE (ranging from 7.6 to 10.6 ng/mL) and PIB (ranging from 8.8 to 13.2 ng/mL) during weekly treatment visits). Figure 2 shows the SVR_12_ rates and 95% CI for the ITT population of all groups.

### Analysis of resistance

Prevalence of baseline polymorphisms in HCV from 10 of the 12 patients with renal impairment is shown in Supporting Table 2 (data not available for 2 patients). Baseline polymorphisms were not detected in NS3, while baseline NS5A polymorphisms were detected in 7/10 patients.

The prevalence of baseline polymorphisms was high among the 32 DAA-experienced GT1b-infected patients; NS3 polymorphisms at position D168 were present in 48.4% (15/31), NS5A polymorphisms at position L31 were present in 81.3% (26/32), and NS5A Y93H was present in 59.4% (19/32) (Table 3). Of these, 41.9% (13/31) had an NS3 D168 polymorphism in combination with an NS5A L31 polymorphism or NS5A Y93H, and 59.4% (19/32) had an NS5A L31 polymorphism with NS5A Y93H. Baseline polymorphisms at two or more of the amino acid positions 28, 30, 31, 32, or 93 in NS5A were detected in 84.4% (27/32) of patients. Baseline polymorphisms had no impact on SVR_12_.

**Table 3:** Prevalence of baseline polymorphisms in DAA-experienced GT1b-infected patients

Target	Baseline polymorphisms^a^	Prevalence, % (*n*/*N*)^b^
NS3	Any^c^	48.4 (15/31)
	D168E/T/V	48.4 (15/31)
NS5A	Any^c^	93.8 (30/32)
	Any ≥ 2^c^	84.4 (27/32)
	L28I/M/T/V	25.0 (8/32)
	R30H/L/M/Q	34.4 (11/32)
	L31F/I/M/V	81.3 (26/32)
	P32deletion	6.3 (2/32)
	Y93F/S	6.3 (2/32)
	Y93H	59.4 (19/32)
NS3 + NS5A	Any NS3 + NS5A^d^	48.4 (15/31)

*NS3* nonstructural viral protein 3, *NS3* ***/*** *4A* nonstructural viral protein 3**/**4A, *NS5A* nonstructural viral protein 5A

^a^The following are considered key amino acid positions: 155, 156, 168 in NS3; and 28, 30, 31, 32, 93 in NS5A. Polymorphisms relative to subtype specific prototypic reference sequences are listed

^b^ *n* = number of patients with baseline polymorphisms at 15% NGS detection threshold; *N* = total number of patients with baseline sequence

^c^“Any” indicates total number of patients with any polymorphism at key amino acid positions within each target gene. Total number of sequences may vary for each target. “Any ≥ 2” indicates the number of patients with baseline NS5A polymorphisms at two or more amino acid positions

^d^“NS3 + NS5A” indicates the total number of patients with baseline polymorphisms in NS3, as well as NS5A and includes only the patients for whom both NS3 and NS5A sequences were available

In this study, 30/32 GT1b-infected patients had previously received asunaprevir + daclatasvir. Two of these 30 (6.7%) patients had P32 deletion in NS5A at baseline; both of these patients experienced virologic failure. P32 deletion in NS5A confers > 1000-fold resistance to PIB. Treatment-emergent substitutions A156D/V in NS3 were detected in one of the two patients who had virologic failure (D168V was also present at baseline and post-baseline in this patient). In NS5A, P32 deletion and L31F/P32 deletion were each present in one of the virologic failure patients at both baseline and post-baseline (Supporting Table 3). The single GT2-infected DAA-experienced patient achieved SVR_12_.

Two GT3-infected patients experienced virologic failure, one infected with GT3b and one infected with GT3k (Supporting Table 3). Twelve GT3-infected patients were enrolled in this study and included diverse subtypes (7 GT3a, 4 GT3b, 1 GT3k). One of the two patients with GT3 infection who failed to achieve SVR_12_ had unusually low plasma concentrations of G/P. Therefore, the impact of baseline polymorphisms on treatment outcome in GT3-infected patients could not be meaningfully assessed.

### Pharmacokinetic results

Following administration of G/P, GLE and PIB plasma concentrations attained steady state by Week 1 visit and remained constant throughout the treatment period (Week 1–Week 8 for non-cirrhotic patients with severe renal impairment with or without dialysis and Week 1–Week 12 for DAA-experienced or cirrhotic patients with severe renal impairment or patients with GT3 infection). The binned geometric mean trough plasma concentrations of GLE and PIB in these subjects are shown in Supporting Table 4. GLE and PIB plasma concentrations were comparable between the 8- and 12-week treatment durations irrespective of prior treatment history or the presence of severe renal impairment. No apparent drug accumulation was observed. GLE plasma concentrations were higher in patients with compensated cirrhosis compared to patients without cirrhosis across all study populations while PIB plasma concentrations were comparable between patients with and without cirrhosis.

### Safety outcomes

TEAEs were experienced by 64%, 83%, and 67% of patients in the DAA-experienced, severe renal impairment and GT3 cohorts, respectively, with 33%, 42%, and 33% assessed as study drug related by investigator. No patient discontinued study drug due to adverse events. One patient with severe renal impairment on hemodialysis experienced an SAE (fluid overload) that was assessed by the investigator as not being study-drug related. TEAEs that occurred at a frequency ≥ 10% in any single cohort included nasopharyngitis, headache, pruritus, abdominal distension, rash, blood creatinine increase, and arthralgia (Table 4).

**Table 4:** Number and percentage of patients with treatment-emergent AEs

Event, *n* (%)	DAA-failures^a^ *N* = 33	Severe renal impairment^b^ *N* = 12	HCV GT3-infected^c^ *N* = 12
Any AE	21 (64)	10 (83)	8 (67)
Any drug-related AE	11 (33)	5 (42)	4 (33)
Any serious AE	0	1 (8)^d^	0
Any study-drug related serious AE	0	0	0
Any AE leading to D/C of study drug	0	0	0
Common AEs (occurring in ≥ 10% in any group)			
Nasopharyngitis	1 (3)	1 (8)	3 (25)
Headache	4 (12)	1 (8)	2 (17)
Pruritus	3 (9)	2 (17)	2 (17)
Abdominal distension	0	0	2 (17)
Rash	1 (3)	1 (8)	2 (17)
Blood creatinine increased	0	2 (17)	0
Arthralgia	0	2 (17)	0

^a^Included GT1- or GT2-infected patients with or without compensated cirrhosis who failed prior DAA treatment

^b^Included GT1- or GT2-infected patients with or without compensated cirrhosis and with severe renal impairment

^c^Included GT3-infected patients with or without compensated cirrhosis

^d^Fluid overload in a patient on dialysis

Laboratory abnormalities were rare across all treatment groups. No grade ≥ 3 elevations (worsening from baseline) occurred in hemoglobin, alanine aminotransferase (ALT), aspartate aminotransferase (AST), or total bilirubin levels (Table 5).

**Table 5:** Post-baseline laboratory abnormalities

Laboratory abnormalities^a^, *n* (%)	DAA-failures^b^ *N* = 33	Severe renal impairment^c^ *N* = 12	HCV GT3-infected^d^ *N* = 12
Hemoglobin			
Grade 2 (< 10–8 g/dL)	1 (3)	0	1 (8)
Grade ≥ 3 (< 8 g/dL)	0	0	0
Alanine aminotransferase			
Grade 2 (> 3–5 × ULN)	0	0	0
Grade ≥ 3 (> 5 × ULN)	0	0	0
Aspartate aminotransferase			
Grade 2 (> 3–5 × ULN)	0	0	1 (8)
Grade ≥ 3 (> 5 × ULN)	0	0	0
Total bilirubin			
Grade 2 (> 1.5–3 × ULN)	2 (6)	1 (8)	0
Grade ≥ 3 (> 3 × ULN)	0	0	0

*ULN* upper limit of the normal range

^a^Grade of event must be more extreme than baseline grade

^b^Included GT1- or GT2-infected patients with or without compensated cirrhosis who failed prior DAA treatment

^c^Included GT1- or GT2-infected patients with or without compensated cirrhosis and with severe renal impairment

^d^Included GT3-infected patients with or without compensated cirrhosis

## Discussion

In this analysis, Japanese patients with limited treatment options: prior DAA-treatment experience, severe renal impairment, or GT3 HCV-infection, achieved SVR_12_ rates of 93.9%, 100%, and 83.3%, respectively, following 8 or 12 weeks of G/P treatment. Four patients experienced virologic failure, including two DAA-experienced and two GT3-infected patients. The two DAA-experienced GT1b-infected patients were previously treated with asunaprevir + daclatasvir and both had P32 deletion in NS5A at baseline. P32 deletion in NS5A has been observed as an uncommon treatment-emergent substitution in patients who have been treated with a daclatasvir-containing regimen [19, 20]. In this study P32 deletion was detected in two of 30 patients who were previously experienced to this regimen. Among DAA-experienced patients, the primary baseline polymorphisms in NS3 and/or NS5A, including those at amino acid position 168 in NS3 or at positions 31 or 93 in NS5A, had no impact on SVR_12_. Of the two GT3-infected patients who had virologic failure, one was treatment-naive and one was pegIFN/RBV-experienced. Plasma concentrations of GLE and PIB were very low throughout treatment in the latter patient without a plausible medical explanation.

The G/P regimen was well-tolerated with no drug-related SAEs reported, no discontinuations of study drug due to AE, and no grade ≥ 3 laboratory abnormalities in hemoglobin, ALT, AST or bilirubin levels observed. Three patients experienced Grade 2 total bilirubin elevations, though none had concurrent ALT elevation or other laboratory abnormality indicative of liver disease progression, and all cases recovered without intervention. Additionally, there were no cases of hepatic decompensation, and no events consistent with drug-induced liver injury were reported. The low rates of laboratory abnormalities observed in this study were also observed in Japanese patients with GT 1 or 2 infection without cirrhosis and with compensated cirrhosis enrolled in the CERTAIN studies and treated with G/P (reported elsewhere [17, 18]). Similar overall safety results were observed across all phase 2 and phase 3 studies conducted outside Japan. The favorable safety profile across all cohorts may enable G/P to be used in broad patient populations, including HCV GT2–6-infected patients with severe renal impairment (CKD 4 and 5; eGFR < 30 mL/min/1.73 m^2^), who currently have no treatment options.

Results from this study are encouraging in light of the currently available treatment options in Japan for the patient populations included in this analysis. According to JSH guidelines, retreatment options are limited for GT1 patients who fail to achieve an SVR with a PI + NS5A inhibitor; moreover, resistance testing is required to select an appropriate regimen. There is no treatment regimen available for GT2–6-infected patients with severe renal impairment, and the only treatment regimen available for GT3–6 patients is 24 weeks in duration and requires RBV [11]. Both DAA-containing regimens currently approved for treatment of Japanese patients with HCV GT2 infection require co-administration of RBV which is contraindicated in patients with moderate or severe renal impairment. Studies of G/P conducted outside Japan have shown high SVR_12_ rates for patients who failed prior DAA therapy [21], patients with severe renal impairment (including those receiving hemodialysis) [22], and treatment-naive patients with GT3 [23]. The findings of this study in Japanese patients are consistent with studies conducted in non-Japanese patients.

In summary, high SVR_12_ rates were achieved with G/P in patients with limited treatment options in Japan, including those with prior DAA experience (93.9%), those with severe renal impairment (100%), and those with GT3 HCV infection (83.3%), and treatment was well-tolerated. These results demonstrate the high efficacy and favorable safety and tolerability of G/P for Japanese patients, including those who currently have limited treatment options.

## Electronic supplementary material

Below is the link to the electronic supplementary material.

Supplementary material 1 (DOCX 100 kb)

## Abbreviations

AE: Adverse event
ALT: Alanine aminotransferase
AST: Aspartate aminotransferase
ASV: Asunaprevir
CI: 95% confidence interval
CKD: Chronic kidney disease
CTCAE: Common Terminology Criteria for Adverse Events
C_trough_: Steady-state trough levels
DAA: Direct acting antiviral agents
DCV: Daclatasvir
eGFR: Estimated glomerular filtration rate
GT: Genotype
GLE: Glecaprevir
G/P: Glecaprevir/pibrentasvir
HCV: Hepatitis C virus
IFN: Interferon
INR: International normalized ratio
ITT: Intent-to-treat
JSH: Japan Society of Hepatology
LLOD: Lower limit of detection
LLN: Lower limit of normal
LLOQ: Lower limit of quantification
NA: Not applicable
pegIFN: Pegylated IFN
PI: Protease inhibitor
PIB: Pibrentasvir
PTW: Post-treatment week
RBV: Ribavirin
SAE: Serious AE
SD: Standard deviation
SMV: Simeprevir
SOF: Sofosbuvir
SVR: Sustained virologic response
TEAE: Treatment-emergent AE
ULN: Upper limit of normal
