# Correction to: Efficacy and safety of glecaprevir/pibrentasvir in HCV-infected Japanese patients with prior DAA experience, severe renal impairment, or genotype 3 infection

**DOI:** 10.1007/s00535-017-1409-z

**Published:** 2017-11-13

**Authors:** Hiromitsu Kumada, Tsunamasa Watanabe, Fumitaka Suzuki, Kenji Ikeda, Ken Sato, Hidenori Toyoda, Masanori Atsukawa, Akio Ido, Akinobu Takaki, Nobuyuki Enomoto, Koji Kato, Katia Alves, Margaret Burroughs, Rebecca Redman, David Pugatch, Tami J. Pilot-Matias, Preethi Krishnan, Rajneet K. Oberoi, Wangang Xie, Kazuaki Chayama

**Affiliations:** 1 Department of Hepatology, Toranomon Hospital, Tokyo, Japan; 2 Department of Internal Medicine, St. Marianna University School of Medicine, Kawasaki, Japan; 3 Department of Medicine and Molecular Science, Gunma University Hospital, Maebashi, Japan; 4 Department of Gastroenterology, Ogaki Municipal Hospital, Gifu, Japan; 5 Department of Internal Medicine, Nippon Medical School, Tokyo, Japan; 6 Department of Human and Environmental Sciences, Kagoshima University Hospital, Kagoshima, Japan; 7 Department of Gastroenterology and Hepatology, Okayama University Graduate School of Medicine, Dentistry, and Pharmaceutical Sciences, Okayama, Japan; 8 The First Department of Internal Medicine, Faculty of Medicine, University of Yamanashi, Yamanashi, Japan; 9 AbbVie Inc., North Chicago, IL USA; 10 Department of Gastroenterology and Metabolism, Institute of Biomedical and Health Sciences, Hiroshima University Hospital, Hiroshima, Japan

## Correction to: J Gastroenterol DOI 10.1007/s00535-017-1396-0

Unfortunately, in the original publication of this article, the copyright line was incorrectly published in PDF as “© The Author(s) 2017” instead of “©The Author(s) 2017 This article is an open access publication” and also the CC-BY description was not included. The description should be as follows.

This article is distributed under the terms of the Creative Commons Attribution 4.0 International License (http://creativecommons.org/licenses/by/4.0/), which permits unrestricted use, distribution, and reproduction in any medium, provided you give appropriate credit to the original author(s) and the source, provide a link to the Creative Commons license, and indicate if changes were made.

The original article was corrected.

