# Supplementary material for: Efficacy and safety of glecaprevir/pibrentasvir in HCV-infected Japanese patients with prior DAA experience, severe renal impairment, or genotype 3 infection
Source: J Gastroenterol. 2017 Oct 20;53(4):566–75. doi: 10.1007/s00535-017-1396-0 (PMC5866827; doi:10.1007/s00535-017-1396-0)
Supplement: Supplementary file 1 — Supplementary material 1 (DOCX 100 kb) [file 535_2017_1396_MOESM1_ESM.docx]

# Online Supporting Information

# Efficacy and Safety of Glecaprevir/Pibrentasvir in HCV-infected Japanese Patients with Prior DAA Experience, Severe Renal Impairment or Genotype 3 Infection

Hiromitsu Kumada, Tsunamasa Watanabe, Fumitaka Suzuki, Kenji Ikeda, Ken Sato, Hidenori Toyoda, Masanori Atsukawa, Akio Ido, Akinobu Takaki, Nobuyuki Enomoto, Koji Kato, Katia Alves, Margaret Burroughs, Rebecca Redman, David Pugatch, Tami J. Pilot-Matias, Preethi Krishnan, Rajneet K. Oberoi, Wangang Xie, Kazuaki Chayama

## Table of contents

[Online Supporting Information 1](#_Toc487126863)

[Efficacy and Safety of Glecaprevir/Pibrentasvir in HCV-infected Japanese Patients with Prior DAA Experience, Severe Renal Impairment or Genotype 3 Infection 1](#_Toc487126864)

[Table of contents 1](#_Toc487126865)

[Eligibility Criteria 2](#_Toc487126866)

[Inclusion 2](#_Toc487126867)

[Exclusion 4](#_Toc487126868)

[Supporting Figure 1. Patient disposition. 6](#_Toc487126869)

[Supporting Table 2. Prevalence of Baseline Polymorphisms in Renally Impaired Patients. 7](#_Toc487126870)

[Supporting Table 3. Baseline polymorphisms and treatment-emergent substitutions in patients experiencing virologic failure 8](#_Toc487126871)

[Supporting Table 4. Binned geometric mean trough plasma concentrations of GLE and PIB 9](#_Toc487126872)

## Eligibility Criteria

### Inclusion

1. Japanese male or female subjects at least 18 years of age at time of screening.

2. Female who is not of childbearing potential or of childbearing potential and sexually active with male partner(s) and currently using at least one effective method of birth control at the time of screening and agrees to practice one effective method of birth control while receiving study drugs starting with Screening and for 30 days after stopping study drug.

3. Sexually active males must be surgically sterile, or if sexually active with female partner(s) of childbearing potential must agree to practice one effective form of birth control starting with Screening and through 30 days after completion of the study drug.

4. Screening central laboratory result indicating HCV single genotype infection for the appropriate treatment arm, without co-infection of any other genotype.

5. Subject has positive anti-HCV Ab and plasma HCV RNA viral load ≥ 1000 IU/mL at Screening Visit.

6. Chronic HCV infection defined as one of the following:

● Positive for anti-HCV antibody (Ab) and/or HCV RNA at least 6 months before Screening.

● A liver biopsy consistent with chronic HCV infection.

7. Subject must be:

● HCV DAA treatment-naïve (i.e., patient has not received a single dose of any approved or investigational DAA). Prior HCV treatment using IFNs with or without ribavirin, is acceptable. Previous HCV IFN based treatment must have been completed ≥ 2 months prior to screening.

**OR**

● HCV DAA treatment experienced (experienced with any approved, commercially available HCV DAA treatment in Japan). Previous HCV DAA treatment must have been completed ≥ 2 months prior to screening (Substudy 2).

8. Must voluntarily sign and date an informed consent form, approved by an Institutional Review Board (IRB)/Independent Ethics Committee (IEC) prior to the initiation of any screening or study specific procedures.

9. Subjects must be able to understand and adhere to the study visit schedule and all other protocol requirements.

In addition to Inclusion Criteria 1 through 9, subjects without cirrhosis must meet the following criteria:

10. Subject must be documented as non-cirrhotic, defined as meeting one of the following criteria:

● A liver biopsy within 24 months prior to or during screening demonstrating the absence of cirrhosis, e.g., a METAVIR, Batts-Ludwig, Knodell, IASL, Scheuer, New Inuyama or Laennec fibrosis score of ≤ 3, Ishak fibrosis score of ≤ 4;

● A FibroScan® score of < 12.5 kPa within 6 months of Screening or during the Screening Period;

● A screening FibroTest score of ≤ 0.72 and Aspartate Aminotransferase to Platelet Ratio Index (APRI) ≤ 2;

● A screening Discriminant Score (z) less than zero, according to the following formula: z = 0.124 × [gamma-globulin (%)] + 0.001 × [hyaluronate (μg × l^–1^)] –0.075 × [platelet (× 10^4^ cells/mm^3^)] – 0.413 × gender (male, 1; female, 2) – 2.005.

In addition to Inclusion Criteria 1 through 9, subjects with compensated cirrhosis must meet the following criteria:

11. Subject must be documented as cirrhotic, defined as meeting one of the following criteria:

● A liver biopsy within 24 months prior to or during screening demonstrating the presence of cirrhosis, e.g., a METAVIR, Batts-Ludwig, Knodell, IASL, Scheuer, New Inuyama fibrosis score > 3 (including 3 – 4 or 3/4), or Laennec fibrosis score of > 3, Ishak fibrosis score of > 4;

● A FibroScan® score of ≥ 14.6 kPa within 6 months of Screening or during the Screening Period;

● A screening FibroTest score of ≥ 0.73 and Aspartate Aminotransferase to Platelet Ratio Index (APRI) > 2;

● A screening Discriminant Score (z) greater than zero, according to the following formula: z = 0.124 × [gamma-globulin (%)] + 0.001 × [hyaluronate (μg × l^–1^)] –0.075 × [platelet (× 10^4^ cells/mm^3^)] – 0.413 × gender (male, 1; female, 2) – 2.005.

12. Absence of hepatocellular carcinoma (HCC) as indicated by an ultrasound, computed tomography (CT) scan or magnetic resonance imaging (MRI) showing no evidence of HCC within 3 months prior to Screening or an ultrasound with no evidence of HCC at Screening. Subjects who have an ultrasound with results suspicious of HCC followed by a subsequent CT or MRI with no evidence of HCC will be eligible for the study.

In addition to Inclusion Criteria 1 through 12, subjects with severe renal impairment (Substudy 2) must meet the following criteria:

13. Subject must have severe renal impairment with estimated glomerular filtration rate (eGFR) < 30 mL/min/1.73 m^2^ (using the MDRD method modified for Japanese population: eGFRJ = 194 × Serum Creatinine^–1.094^ × Age^–0.287^ × 0.739 [if female]), including end-stage renal disease on dialysis.

### Exclusion

1. Female who is pregnant, planning to become pregnant during the study, or breastfeeding; or male whose partner is pregnant or planning to become pregnant during the study.

2. Recent (within 6 months prior to study drug administration) history of drug or alcohol abuse that could preclude adherence to the protocol in the opinion of the investigator.

3. Positive test result at Screening for hepatitis B surface antigen (HBsAg) or anti human immunodeficiency virus antibody (HIV Ab).

4. Requirement for and inability to safely discontinue contraindicated medications or supplements at least 2 weeks or 10 half-lives (whichever is longer) prior to the first dose of any study drug.

5. Clinically significant abnormalities, other than HCV-infection, based upon the results of a medical history, physical examination, vital signs, laboratory profile, and a 12-lead electrocardiogram (ECG) that make the subject an unsuitable candidate for this study in the opinion of the investigator, including, but not limited to:

● Uncontrolled diabetes as defined by a glycated hemoglobin (hemoglobin A1C) level > 8.5% at the Screening Visit.

● Active or suspected malignancy or history of malignancy (other than basal cell skin cancer or cervical carcinoma in situ) in the past 5 years, or any history of HCC.

● Uncontrolled cardiac, respiratory, gastrointestinal, hematologic, neurologic, psychiatric, or other medical disease or disorder, which is unrelated to the existing HCV infection.

6. Any cause of liver disease other than chronic HCV-infection, including but not limited to the following:

● Hemochromatosis, alpha-1 antitrypsin deficiency, Wilson's disease, autoimmune hepatitis, alcoholic liver disease, or steatohepatitis considered to be the primary cause of the liver disease rather than concomitant/incidental with HCV infection.

7. History of solid organ transplantation.

8. Receipt of any investigational product within a time period equal to 10 half-lives of the product, if known, or a minimum of 6 weeks (whichever is longer) prior to study drug administration.

9. Consideration by the investigator, for any reason, that the subject is an unsuitable candidate to receive G/P.

10. History of severe, life-threatening or other significant sensitivity to any study drugs or their excipients.

11. Patients who can't participate in study per local law.

12. Any current or past clinical evidence of Child-Pugh B or C classification or clinical history of decompensated liver disease such as ascites noted on physical exam, hepatic encephalopathy or variceal bleeding.

13. Screening laboratory analyses showing any of the following abnormal laboratory results:

● Estimated glomerular filtration rate (eGFRJ): < 30 mL/min/1.73 m^2^ (For all subjects with the exception of severe renal impairment subjects in Substudy 2)

● Albumin: < LLN for non-cirrhotics, < 2.8 g/dL for cirrhotics

● International normalized ratio (INR): ≥ 1.2 for non-cirrhotics, ≥ 1.8 for cirrhotics (Subjects with a known inherited blood disorder and INR ≥ 1.2 may be enrolled with permission of the AbbVie TA MD.)

● Hemoglobin: < 10 g/dL

● Platelets: < 90,000 cells per mm^3^ for non-cirrhotics, < 50,000 cells per mm^3^ for cirrhotics

## **Supporting Figure 1.** Patient disposition.
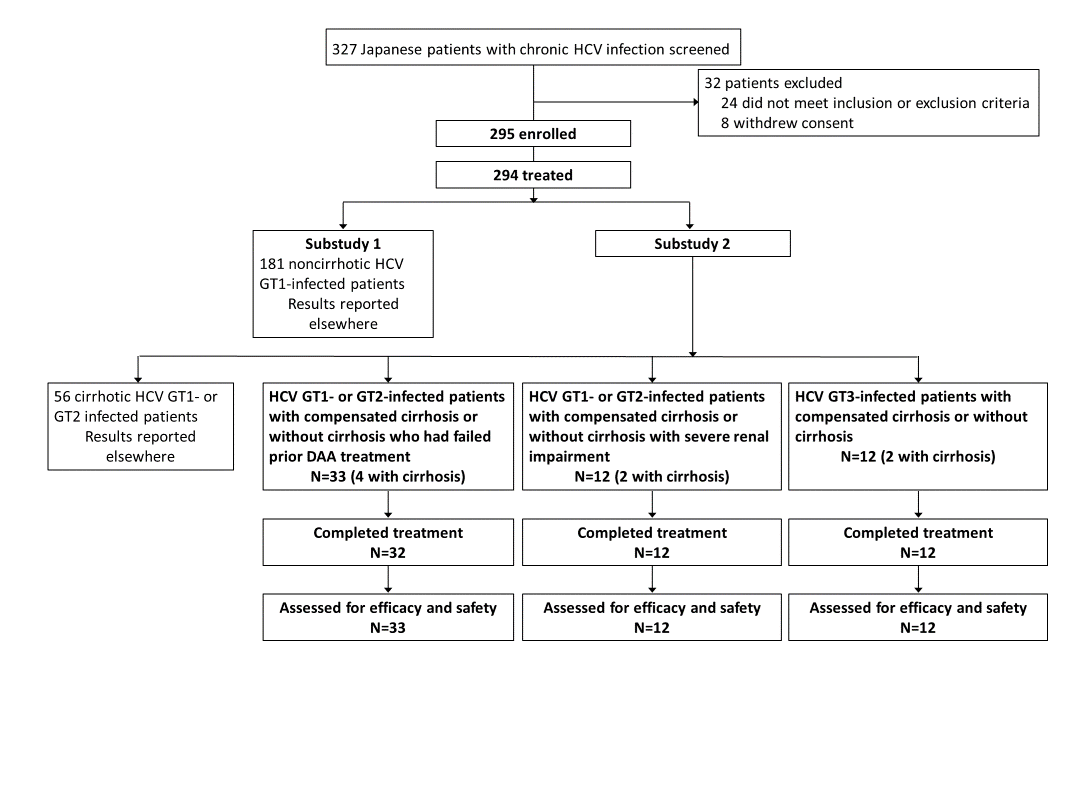


## Supporting Table 2. Prevalence of Baseline Polymorphisms in Renally Impaired Patients.

| **HCV Subtype^a^** | **Target** | **Baseline Polymorphisms^b^** | **Prevalence, % (n/N)**^c^ |
| --- | --- | --- | --- |
| 1b | NS3 | Any^d^ | 0 (0/3) |
|  | NS5A | Any^d^ | 66.7 (2/3) |
|  |  | Y93H | 66.7 (2/3) |
|  | NS3 + NS5A | Any NS3+NS5A^e^ | 0 (0/3) |
| 2a | NS3 | Any^d^ | 0 (0/3) |
|  | NS5A | Any^d^ | 100 (3/3) |
|  |  | L31M | 100 (3/3) |
|  | NS3 + NS5A | Any NS3+NS5A^e^ | 0 (0/3) |
| 2b | NS3 | Any^d^ | 0 (0/4) |
|  | NS5A | Any^d^ | 50.0 (2/4) |
|  |  | L28F | 50.0 (2/4) |
|  | NS3 + NS5A | Any^d^ | 0 (0/4) |

NS3 = nonstructural viral protein 3; NS3**/**4A**=**nonstructural viral protein 3**/**4A; NS5A = nonstructural viral protein 5A

1. Subtype determined by phylogenetic analysis of NS3/4A and/or NS5A baseline sequences. Two patients did not have available subtype; NS3 and NS5A sequences were not available from these 2 patients.
2. The following are considered key amino acid positions: 155, 156, 168 in NS3; and 28, 30, 31, 32, 93 in NS5A. Polymorphisms relative to subtype specific prototypic reference sequences are listed.
3. n = number of patients with baseline polymorphisms at 15% NGS detection threshold; N = total number of patients with baseline sequence.
4. 'Any' indicates total number of patients with any polymorphism at key amino acid positions within each target gene. Total number of sequences may vary for each target.
5. ‘NS3 + NS5A' indicates the total number of patients with baseline polymorphisms in NS3 as well as NS5A, and includes only the patients for whom both NS3 and NS5A sequences were available.

## Supporting Table 3. Baseline polymorphisms and treatment-emergent substitutions in patients experiencing virologic failure

| **Prior HCV Treatment Experience** | **HCV GT** | **Subject Characteristics** | **NS3^a^** | | **NS5A^a^** | | **Treatment outcome** |
| --- | --- | --- | --- | --- | --- | --- | --- |
|  |  |  | **Baseline Polymorphisms** | **Substitutions at Time of VF** | **Baseline Polymorphisms** | **Substitutions at Time of VF** |  |
| NS5A inhibitor  + PI-experienced | 1b | Cirrhotic,  no renal  impairment | D168V | A156D/V, D168V | P32 deletion | P32 deletion | On-treatment virologic failure |
| NS5A inhibitor  + PI-experienced | 1b | Noncirrhotic,  no renal  impairment | None^b^ | None^b^ | L31F,  P32 deletion | L31F,  P32 deletion | Relapse |
| Treatment-naive | 3k | Noncirrhotic,  no renal  impairment | NA | NA | None^b^ | L28F, Y93H | Relapse |
| IFN-treatment experienced | 3b | Noncirrhotic,  no renal  impairment | None^b^ | NA | V31M | V31M, Y93H | Relapse |

GT = HCV subtype by phylogenetic analysis; NA = not available due to technical reasons; PI = protease inhibitor; VF = virologic failure

1. The following are considered key amino acid positions: 155, 156, 168 in NS3; and 28, 30, 31, 32, 93 in NS5A. Polymorphisms relative to subtype specific prototypic reference sequences at 15% detection threshold are listed.
2. 'None' indicates that polymorphisms were not detected at signature amino acid positions.

## Supporting Table 4. Binned geometric mean (%CV) trough plasma concentrations of GLE and PIB for weeks 1–12 or weeks 1–8*

|  |  | GLE (ng/mL) | PIB (ng/mL) |
| --- | --- | --- | --- |
| DAA-experienced | With cirrhosis | 108 (70%) | 59 (72%) |
|  | Without cirrhosis | 26 (180%) | 32 (77%) |
| GT3 | With cirrhosis | 152 (109%) | 58 (37%) |
|  | Without cirrhosis | 26 (72%) | 44 (56%) |
| Severe Renal impairment | With cirrhosis | 51 (100%) | 21 (80%) |
|  | Without cirrhosis^*^ | 58 (233%) | 26 (176%) |

CV, Coefficient of Variation

^*^8 weeks of treatment; all others were treated for 12 weeks with GLE/PIB 300/120 mg once daily
